# Selected abstracts of Bioinformatics: from Algorithms to Applications 2021 Conference

**DOI:** 10.1186/s12859-021-04475-z

**Published:** 2021-12-22

## I1

### Fifth International Conference “Bioinformatics: from Algorithms to Applications” (BiATA 2021)

#### Anton Korobeynikov^1^, Alla Lapidus^1,*^

##### ^1^Center for Algorithmic Biotechnologies, Saint Petersburg State University, Saint Petersburg, Russia, 199034

*Correspondence: Alla Lapidus a.lapidus@spbu.ru

The annual international conference "Bioinformatics: From Algorithms to Applications" (BiATA) was hosted in Saint Petersburg, Russia on July 12–15, 2021 and was accompanied by a two-day online workshop dedicated to metagenomic data analysis.

This year BiATA celebrated its 5th anniversary. Since its inception the conference has quickly gained international renown and prestige by bringing together leading scientists from all over the world. Every year the invited speaker list was invariably filled with top minds in the field.

For all five years, the conference has remained one of the few international events aiming to unite algorithm developers and programmers who create tools for a wide range of research in all areas of modern life science and their users. Keeping this objective in mind, BiATA aims to promote multidisciplinary research and education.

In line with our traditions, BiATA2021 contributes to the active spread of bioinformatics across all areas of life science research, identifies new trends and needs in such important areas of bioinformatics science as algorithmic bioinformatics, genomics, transcriptomics, and the search for biologically active molecules which could prove to be important contributors in the fields of agriculture, medicine and other spheres of human life.

Topics covered by the conference traditionally include, but are not limited to:

- Sequencing technologies
- Assembly of the genome
- Genomic sequence analysis
- Genomics
- Transcriptomics
- Metagenomics
- Natural Products Discovery
- Agrigenomics
- Viromics

As part of the conference’s ongoing collaboration with EMBL-EBI, a two-day online seminar was held once again covering the analysis and annotation of metagenomic data using MGnify [1].

The online format of the conference, which was necessary due to the constantly changing epidemiological restrictions caused by COVID-19, did not hinder the speakers’ ability to present interesting oral and poster presentations.

Abstracts published in this BMC Bioinformatics supplement cover software tools, sequence analysis, genome assembly, comparative genomics and more.

The conference was financially supported by the Ministry of Science and Higher Education of the Russian Federation (№ 075-15-2020-922, 16.11.2020).

**Reference**

1. Mitchell AL, et al., MGnify: the microbiome analysis resource in 2020. Nucleic Acids Res. 2020 Jan 8;48(D1):D570-D578. https://doi.org/10.1093/nar/gkz1035

## O1

### Optimal de novo assemblies for chloroplast genomes based on inverted repeats patterns

#### Rumen Andonov^1*^, Victor Epain^1*^, Dominique Lavenier^1^

##### ^1^Univ Rennes, Inria, CNRS, IRISA, F-35000 Rennes, France

*Correspondence: Rumen Andonov rumen.andonov@irisa.fr; Victor Epain victor.epain@laposte.net

**Background** Chloroplast genome assembly remains challenging because sequencing step outputs short reads both from plant and plastid genomes. Some recent dedicated assemblers [1, 2] use the information of a highly conserved circular and quadripartite structure with a pair of dispersed inverted repeat regions in chloroplast genomes.

**Methods** We designed a dedicated pattern-driven de novo assembler which requires short unpaired reads uniquely (distances provided by paired-reads are not needed), sequenced from both the plant and its chloroplasts. A first step consists in separating the chloroplasts reads from the reads specific to plant. To this end we use the observation that the chloroplast genomes are overrepresented compared to the plant genome. Then we compute an estimated coverage of the pre-assembled contigs and we keep the ones with higher coverage. The first step outputs an assembly graph where each vertex corresponds to a contig and is provided with an estimated multiplicity number. In the sequel we use another graph where each vertex is duplicated according to its multiplicity number and to the two possible contig orientations. The edges are duplicated respectively. In our approach the genome assembly is modeled as finding an elementary path in this graph. We formulate the dispersed repeats as linear constraints and we search for an elementary path using Integer Linear Programming similarly to [3]. In our approach inverted repeats correspond to occurrences of contigs paired with other occurrences of them but in reverse orientation. Their positions on the assembled sequence must satisfy nested-pairs pattern. We formulate the above constraints in terms of linear program where the objective is to maximize the nested-pairs number. Thus, we generalize a similar approach applied for RNA folding [4]. Indeed, in contrast to the later approach where the vertices correspond to bases with known sequence indices, in our case the positions of the contigs are variables. Our tool is implemented with Python 3 and uses the open-source PuLP package which integrates a free solver to solve the above optimization problem.

**Results** We tested our program with QUAST [5] and we obtained very encouraging preliminary results, with high genome coverage (mostly > 99%), and very low mismatches and indels rates.

**Conclusions** We designed a chloroplast genome dedicated pattern-driven de novo assembler using only short unpaired reads. We formulate the conserved circular and quadripartite structure as linear constraints and implemented this model in an open-source program. Finally, QUAST evaluation returned some encouraging preliminary results.

**Acknowledgements **The two first authors have equally participated to the presented work. The first step of the method was designed and implemented by Dominique Lavenier, the second was designed by Rumen Andonov and Victor Epain who implemented it.

**References**

1. Jin J-J, Yu W-B, Yang J-B, Song Y, dePamphilis CW, Yi T-S, et al. GetOrganelle: a fast and versatile toolkit for accurate de novo assembly of organelle genomes. Genome Biology. 2020 Sep 10;21(1):241.
2. Dierckxsens N, Mardulyn P, Smits G. NOVOPlasty: de novo assembly of organelle genomes from whole genome data. Nucleic Acids Res. 2017 Feb 28;45(4):e18.
3. Andonov R, Djidjev H, François S, Lavenier D. Complete assembly of circular and chloroplast genomes based on global optimization. J Bioinform Comput Biol. 2019 Jun;17(3):1950014.
4. Gusfield D. The RNA-Folding Problem. In: Integer Linear Programming in Computational and Systems Biology: An Entry-Level Text and Course. Cambridge: Cambridge University Press; 2019. p. 105–121.
5. Gurevich A, Saveliev V, Vyahhi N, Tesler G. QUAST: quality assessment tool for genome assemblies. Bioinformatics. 2013 Apr 15;29(8):1072–5.

## O2

### BinSPreader: refine binning results for fuller MAG reconstruction

#### Yury Kamenev^1^, Roman Kruglikov^2^, Ivan Tolstoganov^3^, Anton Korobeynikov^3,*^

##### ^1^ITMO University, Saint Petersburg, Russia; ^2^Lomonosov Moscow State University, Moscow, Russia; ^3^Center for Algorithmic Biotechnology, Saint Petersburg State University, Saint Petersburg, Russia

*Correspondence: Anton Korobeynikov a.korobeynikov@spbu.ru

**Background** Despite the recent advances in high-throughput sequencing, analysis of the metagenome of the whole microbial population still remains a challenge. In particular, the metagenome-assembled genomes (MAGs) are often fragmented due to interspecies repeats, uneven coverage and vastly different strain abundance. MAGs are usually constructed via a dedicated binning process that uses different features of input data in order to cluster contigs that might belong to the same species. This process has some limitations and therefore binners usually discard contigs that are shorter than several kilobases. Therefore, binning of even simple metagenome assemblies can miss a decent fraction of contigs and resulting MAGs oftentimes do not contain important conservative sequences. State-of-the-art binners use different kinds of information to cluster contigs but the complete information about the assembly is provided via not the set of contigs but the assembly graph. Nevertheless, recently developed graph-aware binning refining tools such as GraphBin2 and Binnacle do not use the assembly graph in the usual sense of the term. Instead, they are relying on the so-called scaffold graph that only preserves the connectivity information between different scaffolds and loses information about the multiplicity of edges and contig’s edges. In order to utilize this information we suggest using the original assembly graph.

**Methods** Firstly, we use the paths of scaffolds over the assembly graph to mark corresponding edges with labels via input binning. Then bin labels of edges are propagated to neighbors via so-called noisy multi-label semi-supervised clustering with possible correction and bin assignment of all scaffolds is determined from bin labels of edges contained. The resulting most probable set of bins the scaffold belongs to is inferred from the bin distribution of a scaffold. This approach can be used not only for scaffolds, but either for reads to enable fuller MAG reconstruction after the reassembly of subsets of input reads. Besides the assembly graph connectivity BinSPreader is capable of using other kinds of genome connectivity information including paired-end reads, Hi-C links, etc. Utilization of these kinds of input data could further improve the results.

**Results** We tested BinSPreader on mock metagenomes BMock12 and Zymo, comprising 12 and 10 known species, respectively. Shotgun metagenomic assemblies for both samples were obtained using SPAdes v3.15.2. Initial MAGs were produced using MetaBAT2. Estimated with AMBER v2.0.2. average MAG F1 score in base pairs increased from 0.601 in initial MAGs to 0.695 in the same MAGs which were refined by BinSPreader for BMock12 and from 0.731 to 0.802 for Zymo.

**Conclusion** We have shown that BinSPreader could effectively improve the binning increasing the completeness of the bins without sacrificing the purity and could predict contigs belonging to several MAGs. Read splitting that takes into account possible overlap between MAGs enables fuller MAG reconstruction after reassembly.

**Acknowledgements** This work was supported by the Russian Science Foundation (grant 19-14-00172). The research was carried out in part by computational resources provided by the Resource Center “Computer Center of SPbU”. The authors are grateful to Saint Petersburg State University for the overall support of this work.

## O3

### Reconstructing the genome-wide map of relative background allelic dosage with bayesian changepoint identification from read coverage at heterozygous single-nucleotide variants

#### Alexandr Boytsov^1,2^, Sergey Abramov^1,2^, Ivan V. Kulakovskiy^1,3,4*^, Vsevolod J. Makeev^1,2,5*^

##### ^1^Vavilov Institute of General Genetics, Russian Academy of Sciences, Gubkina 3, Moscow, GSP-1, 119991, Russia; ^2^Moscow Institute of Physics and Technology, Institutskiy per. 9, Dolgoprudny, 141700, Russia; ^3^Institute of Protein Research, Russian Academy of Sciences, Institutskaya 4, Pushchino, 142290, Russia; ^4^Center for Precision Genome Editing and Genetic Technologies for Biomedicine, Engelhardt Institute of Molecular Biology, Russian Academy of Sciences, Vavilova 32, Moscow, 119991, Russia; ^5^State Research Institute of Genetics and Selection of Industrial Microorganisms of the National Research Center Kurchatov Institute, Moscow, Russia

*Correspondence: Vsevolod J. Makeev vsevolod.makeev@vigg.ru, Ivan V. Kulakovskiy ivan.kulakovskiy@gmail.com

**Background** Aneuploidy and copy number variants substantially contribute to genetic variation, change gene dosage, and are often associated with pathological conditions. Rapidly advancing DNA sequencing technologies make it possible to infer genomic copy numbers of particular alleles. This is an important stage for computational discovery of allele-specific events, including allele-specific gene expression, chromatin accessibility, and transcription factor binding. However, evaluation of genomic copy numbers usually relies on the whole-genome sequencing data, particularly, long reads and deep sequencing are necessary for reliable haplotype reconstruction. Quite often there are no whole-genome data suitable to reconstruct the genome copy-number profiles for a particular cell type under study. Yet, in gene regulation studies it is often enough to know only the relative dosage of alternative alleles rather than their exact copy numbers.

**Methods **The algorithm utilizes the marginal likelihood segmentation of the genome into regions with approximately constant allele dosage ratio at heterozygous SNVs, while BAD is assigned to each region according to the maximum posterior. We applied BABACHI to more than 15,000 open-source ChIP-Seq and more than 2000 DNAse-Seq read alignments, uniformly processed in the Gene Transcription Regulation Database [1] and validated the algorithm performance with allele-specific copy number profiles from COSMIC database [2]. The SNV-level Kendall-tau b rank correlation was used as the similarity measure between the COSMIC and BABACHI BAD profiles.

**Results** Here we present a novel algorithm, BABACHI (reconstructing Background Allelic Dosage with Bayesian Checkpoint Identification), for efficient inference of whole-genome profiles of the relative background allelic dosage (BAD). These BAD profiles are reconstructed directly from heterozygous variant calls and are consistent if obtained from whole-genome sequencing or from target sequencing data, such as ChIP-Seq or DNase-Seq. We show how BABACHI can be used in analysis of allele-specific transcription factor binding across heterogenous ChIP-Seq datasets, where background allelic dosage explains the overdispersion of the distribution of read counts between alternative alleles.

**Conclusions** We have developed a computationally efficient algorithm to infer the relative allelic copy number imbalance directly from the heterozygous variant calls. In contrast to the existing tools for allele-specific copy number calling, BABACHI does not require neither phased haplotypes, nor whole-genome sequencing data, and allows constructing the genome-wide BAD profiles from enriched sequencing data alone. These profiles can be used as a baseline in statistical evaluation of allele-specific events, such as allele-specific transcription factor binding and allele-specific chromatin accessibility.

**References**

1. Yevshin, I., Sharipov, R., Kolmykov, S., Kondrakhin, Y. & Kolpakov, F. GTRD: a database on gene transcription regulation—2019 update. *Nucleic Acids Res.* 47, D100–D105 (2019).
2. Tate, J. G. et al. COSMIC: the catalogue of somatic mutations in cancer. *Nucleic Acids Res.* 47, D941–D947 (2019).

## O4

### Comparing bioinformatic ASV-level pipelines for microbial V3V4 amplicon 16S rRNA sequencing

#### Irina A. Tsvetkova^1*^, Polina A. Pavlova^2^, Daria V. Likholetova^1^, Vladimir V. Gostev^1,3^, Ekaterina V. Nikitina^1^, Polina S. Chulkova^1^, Vladimir A. Ageevets^1^, Ofelia S. Sulyan^1^, Olga S. Kalinogorskaya^1^, Sergey V. Sidorenko^1,3^

##### ^1^Department of medical microbiology and molecular epidemiology, Federal State-Financed Institution Pediatric Research and Clinical Center for Infectious Diseases under the Federal Medical Biological Agency, Saint-Petersburg, Russia; ^2^ Saint-Petersburg State University, Biology Faculty, Saint-Petersburg, Russia; ^3^North-Western State Medical University named after I.I. Mechnikov, Saint-Petersburg, Russia

*Correspondence: Irina A. Tsvetkova i.tsvetik@gmail.com

**Background** There are several ASV-level pipelines for 16S rRNA-based metagenomic analysis: QIIME2-Deblur, Dada2, Usearch-unoise3. Using different approaches, we can face the problem of loss of compositional reproducibility for the same microbial community. We compared the influence of different parameters of reads processing on the OTUs clustering results.

**Methods** 850 samples, including the unlinked anonymized throat swabs from the laboratory of Department of medical microbiology and molecular epidemiology and the mock community controls (ZymoBIOMICS™) were used for the analysis. 16S V3-V4 metagenomic sequencing libraries were prepared according to standard Illumina protocol. The steps of analysis: reads processing, denoising, chimera filtrarion, getting ASVs; getting ZOTUS with usearch-unoise3 and clustering ASVs into OTUs99% and OTUs97%; OTU abundance table obtaining; assesment of alpha-diversity in the samples; normalization of OTUs abundancies to 11,000 sequences per sample; taxonomic annotation. Strict filtration parameters: Dada2 (minQ = 20, minFoldParentOverAbundance = 1), Dada2 (minQ = 20, minFoldParentOverAbundance = 15). Soft filtration parameters: QIIME2-Deblur (minQ = 0, p-minovlen = 40, p-maxdiffs = 30), Dada2 (minQ = 6, ASVs length filtration: 419–429 bp), Dada2 (minQ = 6, without ASVs length filtration).

**Results** Different percentages of raw reads were saved after denoising and chimera removing: 5.6% and 6.5% for “strict” pipelines; 29%, 36.9% and 42.1% for “soft” pipelines. Comparable number of OTUs97 for different pipeline was received. There were no correlation between ZOTUS alpha-diversity (Shannon index) in samples, before data normalization, for “strict” and “soft” pipelines (Spearman coefficient = 0.22–0.25). OTUs97 and ZOTUS alpha-diversity did not correlate within “strict” pipelines. Almost always, the mock’s taxa ratio had been reproduced well within annotated ZOTUS and OTUs97 for all pipelines. But the optimal SINTAX bootstraping cut-off value in our study was 20 (for annotation with training RDP dataset). We observed a lot of unassigned sequences with bootstraping cut-off = 80. The best reproducibility of ZOTUS and OTUs97 with 100% identity to mock’s reference sequences was observed for Dada2 with soft filtration parameters (minQ = 6, without amplicon length filtration).

**Conclusions** Pipelines performances were evaluated for a large set of samples, a reliable correlation between the alpha-diversity within OTUs97 and ZOTUS clustering, for all samples, was observed for the Dada2 algorithm with soft read filtering parameters. The strict filtering parameters in dada2 lead to a shift in the taxa ratio [1].

**Statement** Ethical committee approval was not required for this study, unlinked anonymized throat swabs from the laboratory were used for the adjustment of bioinformatics methods.

**Reference**

1. Prodan A., et al. Comparing bioinformatic pipelines for microbial 16S rRNA amplicon sequencing. PLoS ONE. 2020; 15(1):e0227434.

## O5

### Testing the EXONtools pipeline: pseudoreference development and hybridization bait design for exon capture experiments

#### Kirill A. Vinnikov^123*^, Kathleen S. Cole^4^

##### ^1^Sirius University of Science and Technology, Sochi, Russia; ^2^Laboratory of Ecology and Evolutionary Biology of Aquatic Organisms, Far Eastern Federal University, Vladivostok, Russia; ^3^Laboratory of Genetics, A.V. Zhirmunsky National Scientific Center of Marine Biology, Far Eastern Branch of the Russian Academy of Sciences, Vladivostok, Russia; ^4^University of Hawaii at Manoa, Honolulu, HI, USA

*Correspondence: Kirill A. Vinnikov vinnikov.ka@dvfu.ru

**Background** Exon capture sequencing approach requires a reference, such as a well-annotated genome or transcriptome, to be used as a template for hybridization baits. These baits resemble short RNA sequences, 80–120 base pairs in length, designed to have a high similarity to their genome target regions that allow for successful gene capture (i.e., hybridization) across all individuals used in a population or phylogenomic study. The EXONtools pipeline is proposed for exon capture sequencing data analysis on non-model species. It includes three major stages: (1) de novo transcriptome assembly and annotation; (2) exon hybridization bait development; and (3) exon–intron loci assembly with ortholog alignment and SNP calling. In this study we examined many possible constraints for the bait design and proposed solutions to how they can be unraveled and processed by using the EXONtools pipeline. Leaving these problems untreated can cause a strong decrease in the bait hybridization efficacy, as well as an appearance of false-positive SNP calls in resulting exon capture data.

**Methods** We tested the EXONtools pipeline performance on several de novo transcriptome assemblies of non-model species from the genus *Stenogobius* (Teleostei: Gobiiformes). The pipeline implements a consensus assembly approach to produce high-quality transcripts that correspond to protein-coding regions, and therefore, can be used as references for exon capture bait design. We named such dataset a “pseudoreference” because it usually does not correspond to any particular individual but represents a combination of various references. Different artifacts present in pseudoreference consensus assemblies were tested, including: (a) incomplete contigs having long gaps (scaffolds); (b) redundant sequences representing the same transcript (isotigs); (c) transcript isoforms produced by alternative splicing events (isoforms); (d) paralogous transcripts resulting from gene duplications (paralogs); and (e) chimeric contigs (misassemblies).

**Results** More than a hundred of different assemblies were produced by three popular assembly programs incorporated in the EXONtools pipeline. Within these assemblies most constraints were successfully discovered and removed. The final *Stenogobius* pseudoreference dataset included > 42 K annotated contigs with predicted CDS regions, including > 25 K unique annotations and > 130 K unique exons. The pseudoreference was used to design hybridization baits for the following exon capture experiments on amphidromous gobies.

**Conclusions** The current study demonstrates how the EXONtools pipeline can be applied for any non-model species of interest to produce hybridization baits with a high capture efficacy. As a universal tool for the development of numerous genome-wide molecular markers, the EXONtools pipeline should boost the performance of phylogenomic and population genomic studies of non-model species to a much higher level.

**Acknowledgements** The reported study was funded by RFBR, project number 19-37-51019 (pipeline development and software testing), and Ministry of Science and Higher Education of the Russian Federation, project number FZNS-2021-0011 (experimental data tests and pipeline approbation).

## O6

### Integrated pipeline for mining biosynthetic gene clusters in microbial metagenomes

#### Laila Ziko^1,2*^, Ahmed Yamany^3^, Lobna A. Ghonaim^3^, Ahmed A. Aboushanab^3^, Hassan Azzazy^4^, Ahmed Moustafa^2,3,5^

##### ^1^School of Life and Medical Sciences, The University of Hertfordshire, hosted by the Global Academic Foundation, Egypt; ^2^Department of Biology, American University in Cairo, Egypt; ^3^Biotechnology Graduate Program, American University in Cairo, Egypt; ^4^Department of Chemistry, American University in Cairo, Egypt; ^5^Bioinformatics and Integrative Genomics Lab, American University in Cairo, New Cairo, Egypt

*Correspondence: Laila Ziko laila.adel@aucegypt.edu

**Background** Specialized metabolites (SMs) with pharmaceutical bioactivities are crucial to study to search for new antibacterial and anticancer agents from nature. One approach to discover new SMs is mining metagenomes of microbes living in uncharted habitats. To comprehensively investigate available microbial metagenomes, we assembled a pipeline to capture both the biosynthetic gene clusters (BGCs) with similarity to known BGCs and novel BGCs.

**Methods** The sequence reads were filtered and assembled into contigs, then the antibiotics & Secondary Metabolite Analysis Shell, antiSMASH 4.0 (Hidden Markov Model [HMM]-based) and DeepBGC (deep learning-based) algorithms were used to detect the BGCs in the assemblies. We processed 118 samples from 10 publicly available microbial metagenomic projects using our pipeline with a total size of ~ 1.5 Gbps.

**Results** 1316 BGCs were detected by antiSMASH in all the environments, while DeepBGC detected 124,118 BGCs. The BGCs identified by antiSMASH were pertaining to 31 different classes, with the largest three groups, Non-ribosomal peptide synthases (NRPSs), terpenes, and bacteriocins. Most DeepBGC-detected BGCs were not assigned specific product classes (80% of the total clusters). Still, instead, they were mainly annotated based on their product activities, such as antibacterial and antifungal activities. Combining the two approaches, we captured more BGCs than using either alone, including novel ones. Here, we focus on the RiPPs class (Ribosomally synthesized and post-translationally modified peptides). Eight different RiPP cluster types were detected by antiSMASH: bacteriocin (Bacteriocin or other unspecified RiPP cluster/RiPP-like), lanthipeptide, LAP (Linear azol(in)e-containing peptides), lasso peptide, RaS-RiPP (Streptide-like thioether-bond RiPPs), sactipeptide, TfuA-related (TfuA-related RiPPs/thioamitides), and thiopeptide. AntiSMASH seems to be more accurate in detecting and annotating the different RiPPs, particularly bacteriocins.

**Conclusions** DeepBGC was advantageous in predicting many more BGCs based on their activities. The two approaches complement each other and enhance the ability to detect the BGCs beyond applying a single method. Our results are expected to be the basis for finding novel bioactive agents from microbes living in diverse ecosystems.

## O7

### S3finder (v. 0.2.1) makes finding taxon-specific sequences easier

#### Polina Rasskazova^1,2*^, Peter Evseev^2^, Konstantin Miroshnikov^2^

##### ^1^Moscow Institute of Physics and Technology, Dolgoprudny, Moskow, Russia; ^2^Shemyakin-Ovchinnikov Institute of Bioorganic Chemistry, Moskow, Russia

*Correspondence: Polina Rasskazova polinchen98@mail.ru

**Background** The use of PCR-based diagnostic systems is one of the most convenient and popular methods for detecting of pathogens. To carry out accurate species-specific diagnostics by PCR analysis, it is important to select a genome region unique for a taxonomic species and primers that exclude nonspecific amplification. A Linux command line pipeline was developed to perform the search for the taxon-specific sequences.

**Methods** The program is implemented in Python using a special Biopython library designed for solving bioinformatics problems. The pipeline employs BLAST program for the similarity analysis.

**Results** The pipeline named S3finder (v. 0.2.1) consists of 3 steps: (1) Data preparation; (2) Search for species-specific areas; (3) Selection of fragments for the construction of PCR primers. At the first step, all genomes of the target genus are downloaded from the NCBI database and two local databases, so called “positive” and “negative” databases, are formed. The positive database comprises the genomes of the strains (representatives) of the target species. The negative database comprises the strains which should not contain target species-specific sequences. At the same step, the genome of the type strain belonging to the “positive” base is selected and sliced into smaller fragments. To implement the first stage, it is necessary to submit the initial data to the program input. The input data are: taxonomic affiliation of target bacterium (type and genus); target genome INSDC; name of working folder. At the second step, the search for unique sequences is carried out. The program receives data from the first stage: the file with sliced genome of target type strain and the “negative” database files. It is possible to set some BLAST parameters such as E-value, the number of processor cores and the type of the search algorithm (blastn, megablast, blastp). The pipeline compares the target genome with the negative database sequences and finds out the similar sequences. After that the program creates two files named as “hits” and “no_hits”, which contain analyzed sequences with similarities to the negative database sequences found (“hits”) and not found (“no_hits”). The next part of the second step is the comparison of the “no_hits” file with the “positive” database genomes to reveal the fragments present in all genomes of the “positive” target groups. At the end of the second step, we get a file with target genome fragments which are specific only for the target group (they have similar regions in all the target positive group genomes and do not have homologous sequences on all the negative group genomes). At the third step, the program works with the file obtained at the second stage concatenating the overlapping sliced fragments found to be taxon-specific. The result of this step is a folder with files each of which contains a region of the target genome that is unique for representatives of the target group. The output files also contain the information about the genomic coordinates of taxon-specific fragments.

**Conclusions** S3Finder is a user-friendly pipeline suitable for finding taxon-specific sequences. It makes S3Finder a convenient instrument for genomic analysis and development of PCR diagnostic systems and genomic analysis.

**Acknowledgements** This research was supported by Russian Science Foundation grant #21-16-00047.

## O8

### PlasPred: computational prediction tool for identifying plastic-degrading microbes

#### Akhil Wilson^1^, Akhil Thankachan^1^, N. Hemalatha^1*^, Lanwin Lobo^2^

##### ^1^Dept of Information Technology & Bioinformatics, St Aloysius College of Management and Information Technology, Mangalore, India; ^2^MResult Services Pvt Ltd, Mangalore, India

*Correspondence: N. Hemalatha hemalatha@staloysius.ac.in

**Background** Plastic pollution has become one of the most stressing environmental issue. It is practically impossible to avoid plastic completely but can degrade these products rather than throwing it into the surroundings. Plastic degradation is required for the successful management of sustainable development. Biological process plays an important role in removing the contaminants and also taking the benefit of microorganisms who are highly versatile to degrade these plastic components [1]. A proper review of previous articles of degrading microbes helped us create a database of biodegradable and non-biodegradable microbes. Here, we have developed a computational prediction model with WEKA, a machine learning tool with various classifiers which will help in predicting degradability of microbes.

**Methods** Plastic degrading protein sequences belonging to the alkB and CYP153 genes were cumulated from databases such as NCBI and UniprotKB. Around 9000 positive protein sequences and 6000 negative sequences were obtained. For training datasets, we divided the datasets in the ratio 60:40. In this work, we have used 6 different features namely amino acid counts, dipeptide counts, amino acid ratio, hydrophobicity, hydrophilicity, acidity and basicity of microbial protein sequences. Count of each amino acid in a protein sequence was divided by its length in the amino acid ratio for which feature dimension was 20. In the dipeptide count, count of the occurrence of all dipeptide was taken and dimension was of size 400. In the physiochemical properties that included hydrophobicity, hydrophilicity, acidity and basicity, feature dimensions were nine, six, two and three simultaneously. WEKA, an open-source machine learning software has been used in this work for practical implementation [2]. Different classifiers used are Sequential Minimal Optimization (SMO), Multi-Layer Perceptron (MLP), J48 a decision tree classifier and Instance Based Learner (IBK).

**Results** Machine learning models were created with all the four classifiers and six different features with our training set. It was found that all the four classifiers successfully classified the microbes with 100% accuracy for amino acid count feature where as for other features it was less than 95%. This feature was also considered the best as it had only a feature dimension of twenty and comparatively less than other features.

**Conclusions** Plastic pollution is a major concern among the scientific community. There are chances that biologists may come across microbes which will have the potential of degradability. To conclude, this model can be used in cases where biologists want to test the degradability of microbes.

**Acknowledgements** This work is a part of the major research project funded by St. Aloysius Management.

**References**

1. Caruso, Gabriella. Plastic degrading microorganisms as a tool for bioremediation of plastic contamination in aquatic environments. J Pollut Eff Cont. 2015; 3(3): 1–2.
2. Holmes G, Donkin A, Witten I H.. Weka: A machine learning workbench. Proceedings of ANZIIS'94-Australian New Zealnd Intelligent Information Systems Conference. IEEE Explore, 1994; 357–361.

## O9

### A simple tool for positional view of results generated by comparative genomic analysis platforms of prokaryotes

#### Romeu C. Z. Luz^1*^, Janira Prichula^2^, Rafaella S. Bueno^1^, Robson D. Ruiz^1^, Ícaro M. S. Castro^3^, Pedro A. d’Azevedo^2^, Ana P.G. Frazzon^4^, and Adriana Seixas^5^

##### ^1^Biomedical Informatic Undergraduate Student, Federal University of Health Sciences of Porto Alegre, Sarmento Leite, 245, Porto Alegre, Rio Grande do Sul (RS), Brazil; ^2^Gram-positive Cocci Laboratory, Federal University of Health Sciences of Porto Alegre (UFCSPA), Porto Alegre, RS, Brazil; ^3^ Institute of Mathematics and Statistics (IME), University of São Paulo (USP), São Paulo, SP, Brazil; ^4^ Department of Microbiology, Federal University of Rio Grande do Sul, Porto Alegre, RS, Brazil; ^5^Department of Pharmacosciences, Federal University of Health Sciences of Porto Alegre, Porto Alegre, RS, Brazil

*Correspondence: Romeu C. Z. Luz romeulu@ufcspa.edu.br

**Background** Genomic characterization is an essential step in understanding the pathogenic potential of a microorganism. In this process, different platforms for comparative biological data analysis have been used. Summarizing findings is often laborious and time-consuming and requires significant data manipulation to better understand the relationships among different results. Here, we present a tool that offers a visual summary of results obtained in comparative analysis platforms used for studies of prokaryote genomes.

**Methods** A search in the literature was performed to select the tools used for the characterization of prokaryotic genomes, specifically those dedicated to identify virulence factors and genetic mobile elements. To build the client interface, we used Electron JS (https://www.electronjs.org/) using JavaScript, HTML, and CSS. In the contents of each analysis tools output file, data position is collected, organized, and written in JavaScript Object Notation (JSON) format in order to guide the construction of the image from these positional references. Creation of the integrative image was greatly facilitated by JavaScript D3.js—Data-Driven Documents—a library that enables the manipulation of visual elements using web standards. The tool allows the customization of image attributes through an editing interface. The free open access and the adoption of user-friendly interfaces guided the whole process of product development and design.

**Results** The following platforms were included to generate the image: (i) ResFinder, (ii) VirulenceFinder, (iii) PlasmidFinder, (iv) Phaster, (v) CRISPRCasFinder, (vi) IslandViewer 4. The user can submit the results file (.csv and tsv) from the analysis of bacterial genomes and access a graphic representation correlating the submitted results in a single circle image, thus speeding and improving the interpretation and understanding of these data. Our image tool was conceived as a desktop application, which works with the most widely used operating systems.

**Conclusions** Our tool allows the construction of graphical images focusing on the positioning presentation of resistance and virulence genes, plasmids, phages, CRISPRs, and genomic islands in the prokaryotic genomes investigated. With this tool, we intend to significantly improve the way to present prokaryotic genomic data, contributing to researchers who work in this area.

## O10

### Near-saturating semi-automated inference of bacterial transcription factor binding sites for regulatory network analysis and producer strain construction

#### Pavel Vychyk^1*^, Yevgeny Nikolaichik^1^

##### ^1^Department of Molecular Biology, Belarusian State University, Minsk, Belarus

*Correspondence: Pavel Vychyk p.vychik@gmail.com

**Background** Most bacterial genome annotations lack information about regulatory elements: promoters, terminators, and transcription factor (TF) binding sites (operators). Although in silico approaches exist for inference of all three types of regulatory elements, complete genome-scale operator analysis was impractical until recently. We have previously presented the SigmoID v2 application, which contains the pipeline for genome-wide operator inference in bacterial genomes [1]. The pipeline exploits the idea of critical residue (CR) tags [2]—a set of amino acid residues making specific contacts with DNA bases—as unique identifiers of TF-operator pairs. Here we describe an approach for automated operators annotation for nearly all TFs encoded in a bacterial genome.

**Methods** We have added additional methods to the existing SigmoID application [https://github.com/nikolaichik/SigmoID] to automate genome-wide operator analysis with different scope. Rather than deciding on the best motif for each TF, we add inferred operators to the annotation, visualize all potential operators near the target gene, perform a small-scale verification of the corresponding motifs and thus find possible regulators of the gene of interest much faster. We have also introduced changes to the pipeline leading to saturating whole-genome analysis: (i) analysis of TFs with two DNA-binding domains (typical for, e.g. the AraC family); (ii) handling variable linkers between the recognition helix and additional DNA base contacting residues; (iii) models for non-helix-turn-helix TFs; (iv) structure-based modelling to infer CR tags for TF families with unknown 3D structures.

**Results** With original SigmoID application, one could infer operators for a TF and get an idea about its regulon composition. The inverse problem is often encountered: the gene of interest is annotated in a genome, but its regulators are unknown. This problem is typical for biotechnology projects aiming at increasing the expression of a particular protein via altering the regulation of its gene. In such a situation, finding regulators of a single gene requires checking operators for all transcription factors encoded in the genome. The modified pipeline allows SigmoID to automatically annotate binding sites for over 90% of TFs for *Enterobacterales* making such analysis near-complete. Such annotation still requires human verification, but this can be done quickly if restricted to genes of interest only.

**Conclusions** The proposed approach could be useful for designing strain modifications. CR-tags for additional TF families important for other taxonomic groups could be easily calculated to achieve similar operator inference efficiency.

**References**

1. Selected abstracts of “Bioinformatics: from Algorithms to Applications 2020” conference. BMC Bioinformatics. 2020;21:567, s12859-020-03838-2.
2. Sahota G, Stormo GD. Novel sequence-based method for identifying transcription factor binding sites in prokaryotic genomes. Bioinformatics. 2010;26:2672–7.

## O11

### IDOPS, a profile HMM-based tool to detect pesticidal sequences and compare their genetic context

#### Stefani Díaz-Valerio^1^, Anat Lev Hacohen^1^, Raphael Schöppe^1^ and Heiko Liesegang^1*^

##### ^1^Genomic and Applied Microbiology & Göttingen Genomics Laboratory, Institute of Microbiology and Genetics, Georg-August University of Göttingen, Göttingen, Germany

*Correspondence: Heiko Liesegang hlieseg@gwdg.de

**Background** Biopesticide-based crop protection is constantly challenged by insect resistance. Thus, expansion of available biopesticides is crucial for sustainable agriculture. Although *Bacillus thuringiensis* is the major agent for pesticide bioprotection, the number of bacteria species synthesizing proteins with biopesticidal potential is much higher. The Bacterial Pesticidal Protein Resource Center (BPPRC) offers a database of sequences for the control of insect pests, grouped in structural classes.

**Methods** We developed IDOPS, a tool that detects novel biopesticidal sequences and analyzes them within their genetic environment. The backbone of the IDOPS detection unit is a curated collection of high-quality hidden Markov models that is in accordance with the BPPRC structure-based nomenclature. Gene expression depends on the genomic environment, therefore, IDOPS provides a comparative genomics module to investigate the genetic regions surrounding pesticidal genes.

**Results** IDOPS was positively benchmarked with BtToxin_Digger and Cry_Processor. In addition, a scan of the UniProtKB database using IDOPS models returned an abundance of new pesticidal protein candidates distributed across all the structural groups. The comparative genomics feature enables the investigation of accessory elements and evolutionary traits relevant for optimal toxin expression and functional diversification.

**Conclusions** IDOPS contributes and expands our current arsenal of pesticidal proteins used for crop protection.

## O12

### MMseqs2 profile/profile: fast and ultra-sensitive searches beyond the twilight zone

#### Milot Mirdita^1#^, Hyunjoo Ji^2#^, Eli Levy Karin^1^, Hans-Georg Sommer^1^, Clovis Galiez^3^, Johannes Söding^1*^, Martin Steinegger^2*^

##### ^1^Quantitative Biology and Bioinformatics, Max Planck Institute for Biophysical Chemistry, Göttingen, Germany, ^2^Biological Sciences Department, College of Natural Sciences, Seoul National University, Seoul, South Korea, ^3^Université Grenoble Alpes, CNRS, Grenoble INP/Institute of Engineering Université Grenoble Alpes, Grenoble, France

*Correspondence: Johannes Söding soeding@mpibpc.mpg.de; Martin Steinegger martin.steinegger@snu.ac.kr

^**#**^Authors contributed equally

**Background** Analyses of high-throughput sequencing studies are producing billions of novel, uncharacterized protein sequences of previously unstudied organisms. State-of-the-art sequence-to-sequence alignment [1] methods are well-tuned to cope with the avalanche of data. However, they are limited by their sensitivity as they rely on existing homologous sequences in reference databases within the daylight-zone [2] of detectability. The most sensitive alignment methods, HHblits [3] and HHsearch, are based on HMM to HMM (profile-profile) alignments and are able to detect remote homologies across vast evolutionary distances, well into the midnight-zone. However, they are limited in their applicability since they take minutes to process a single query, despite large optimization efforts. Here, we propose MMseqs2 Profile/Profile, the first profile-profile alignment method to match the sensitivity of HHblits at much higher runtime speed.

**Methods** We extend the fast SIMD-accelerated implementation of the Striped Smith-Waterman-Gotoh algorithm [4] in MMseqs2 to support profile-profile alignments and introduce efficient workflows for reverse- and iterative-searches for the construction of extremely diverse multiple sequence alignments.

**Results** We achieve nearly 13,700 × the speed of HMMer3 [5] while being at most 33% more sensitive, and at nearly 160 × the speed of HHblits we match its sensitivity. Furthermore, we expect MMseqs2 Profile/Profile to scale well onto large query datasets, due to its efficient parallelization.

**Conclusions** MMseqs2 profile/profile is well suited to efficiently detect remote homologs beyond the twilight zone. It will help to annotate a large fraction of proteins of previously unstudied organism. It is open source available at MMseqs2 [https://mmseqs.com].

**Acknowledgements** This work was supported by National Research Foundation of Korea [2019R1A6A1A10073437, 2020M3A9G710393, 2021R1C1C10206]; New Faculty Startup Fund, Creative-Pioneering Researchers Program at the Seoul National University, and the BMBF CompLifeSci project horizontal4meta. E.LK. is a FEBS long-term fellowship recipient.

**References**

1. Rost, B. Twilight zone of protein sequence alignments. *Protein Eng.* 1999; **12**, 85–94.
2. Steinegger, M. et al*.* HH-suite3 for fast remote homology detection and deep protein annotation. *BMC Bioinformatics* 2019; **20**, 473.
3. Steinegger, M. & Söding, J. MMseqs2 enables sensitive protein sequence searching for the analysis of massive data sets. *Nat. Biotechnol.* 2017; **35**, 1026–1028.
4. Farrar, M. Striped Smith-Waterman speeds database searches six times over other SIMD implementations. *Bioinformatics* 2007; **23**, 156–161.
5. Eddy, S. R. Accelerated profile HMM searches. *PLoS Comput. Biol.* 2011 **7**, e1002195.

## P1

### Computational prediction of biological activities of peptidic natural products

#### Alexandra Sadovskaya^1*^, Alexey Gurevich^2^

##### ^1^Department of Biology, Bioinformatics MS program, St. Petersburg State University, St. Petersburg, Russia; ^2^Center for Algorithmic Biotechnology, St. Petersburg State University, St. Petersburg, Russia

*Correspondence: Alexandra Sadovskaya alexandrasad04@gmail.com

**Background** Peptidic Natural Products (PNPs) are a rich source of antibiotics and other pharmaceuticals. Recent breakthroughs in mass spectrometry (MS) and genome sequencing enabled high-throughput PNP discovery. Emerging computational methods allowed identification of novel PNPs via modification-tolerant database search of MS data [1], de novo MS sequencing [2], and metabologenomics approaches [3, 4]. These methods produce large amounts of in silico predicted PNPs that require further experimental analysis. Creating a robust strategy for selecting the most promising compounds from many computationally predicted molecules remains an open problem.

**Methods** We developed a computational pipeline for predicting the biological activities of PNPs based on their chemical structure. The pipeline takes as input molecules in standard chemical formats, converts them into SMILES, and scans public databases for biological activity data associated with the molecules. If the database lacks biological activity data for a molecule, the pipeline queries structurally similar molecules. The pipeline structures all retrieved information into a combined summary report and detailed reports per each input molecule. We implemented the software in Python with the use of the RDKit library.

**Results** Our pipeline accepts PNPs in the SMILES and MDL MOL formats and retrieves biological activities from the PubChem database [5]. We plan to integrate the developing pipeline with the state-of-the-art approaches to PNP discover [1–4] to complement their output with the tentative biological activity of the identified compounds. Support for other databases is scheduled for future releases of the pipeline.

**Conclusions** We anticipate our pipeline will facilitate the search for new therapeutic agents by providing researchers with biologically relevant information. This data will help to prioritize in silico predicted PNPs for experimental validation and testing.

**Acknowledgements** The reported study was supported by the Russian Science Foundation (grant 20-74-00032).

**References**

1. Gurevich A, Mikheenko A, Shlemov A, Korobeynikov A, Mohimani H, Pevzner P. Increased diversity of peptidic natural products revealed by modification-tolerant database search of mass spectra. Nat Microbiol. 2018; 3: 319–327.
2. Behsaz B, Mohimani H, Gurevich A, Prjibelski A, Fisher M, Vargas F, et al. De novo peptide sequencing reveals many cyclopeptides in the human gut and other environments. Cell Syst. 2020; 10: 99–108.
3. Cao L, Gurevich A, Alexander K, Naman C, Leão T, Glukhov E, et al. MetaMiner: a scalable peptidogenomics approach for discovery of ribosomal peptide natural products with blind modifications from microbial communities. Cell Syst. 2019; 9: 600–608.
4. Behsaz B, Bode E, Gurevich A, Shi Y, Grundmann F, Acharya D, et al. Integrating genomics and metabolomics for scalable non-ribosomal peptide discovery. Nat Commun. 2021; 12: 3225.
5. Kim S, Chen J, Cheng T, Gindulyte A, He J, He S, et al. PubChem in 2021: new data content and improved web interfaces. Nucleic Acids Res. 2020; 49: D1388-D1395.

## P2

### Presumable bacterial symbionts of the White Sea marine sponges

#### Anastasiia Rusanova^1,3*^, Dmitry Sutormin^1,2^, Victor Fedorchuk^4^, Margarita Ezhova^5^, Stepan Toshchakov^6^, Svetlana Dubiley^1,2^, Konstantin Severinov^2,3,7^

##### ^1^Institute of Gene Biology, Russian Academy of Sciences, Moscow, 119334, Russia; ^2^Skolkovo Institute of Science and Technology, Moscow, 121205, Russia; ^3^Institute of Molecular Genetics of National Research Centre «Kurchatov Institute», Moscow, 123182, Russia; ^4^Lomonosov Moscow State University, Moscow, 119234, Russia; ^5^A.A. Kharkevich Institute for Information Transmission Problems, Russian Academy of Sciences, Moscow, 127051, Russia; ^6^Kurchatov Center for Genome Research, National Research Center “Kurchatov Institute”, Moscow, 123182, Russia; ^7^Waksman Institute of Microbiology, NJ, 08854, USA

*Correspondence: Anastasiia Rusanova molbiol1970@gmail.com

**Background** Sponges comprise complex microbial communities, which differ in taxonomic composition from those of the surrounding seawater. Modern sequencing technology allows us to characterize the diversity of microorganisms in the communities and predict their properties. Since marine sponges of the cold waters remain less studied compared to those from tropical and subtropical regions, we focused on the specimens collected in the White Sea. In this work, for the first time, we applied a metagenomic approach to the analysis of microbial communities associated with sponges from the Russian Arctic.

**Methods** The samples of three marine sponges *Isodictya palmata*, *Halichondria panicea*, *Halichondria sitiens*, and the surrounding seawater were collected in the Kandalaksha Bay, the White Sea in August 2016 and 2018. Total DNA was extracted from the enriched microbial fraction and sequenced using Illumina NextSeq. Metagenomic reads were assembled using metaSPAdes. MaxBin2 and CONCOCT were used to recover metagenome-assembled genomes (MAGs). Quality control and taxonomy annotation of MAGs were performed with CheckM and GTDB. For annotation of proteins, BlastKOALA and WebMGA were used. For 16S metagenomics, the V3-V4 region of 16S rRNA was amplified and sequenced on Illumina MiSeq. Obtained sequences were analyzed with DADA2 and resulting amplicon sequence variants (ASVs) were clustered in operational taxonomic units (OTUs) using MMseqs2. OTU associated with a host sponge was defined as an OTU abundant in a sponge (frequency > 0.05) and significantly enriched in a sponge over the surrounding seawater (sponge/water > 10).

**Results** From the 16S metagenomic data, we found that microbial communities associated with sponge species were dominated by several sponge-specific OTUs identified in the samples from both 2016 and 2018. From metagenome assemblies, we managed to recover MAGs corresponding to the most abundant sponge-associated OTUs. The MAG from *H. panicea* metagenome was classified as *Amylibacter* and was similar to the genome assembly of a symbiont previously identified for *H. panicea* from Icelandic waters. Distinct sponge-associated MAGs from *I. palmata* and *H. sitiens* metagenomes belonged to Gammaproteobacteria and have not been previously described. In MAGs from *H. panicea* and *H. sitiens* metagenomes, we revealed the presence of vitamin B12 biosynthesis pathway, which may be a symbiotic feature.

**Conclusions** Microbial communities associated with the studied marine sponges from the White Sea harbor prevalent sponge-specific bacteria, that had low or zero abundance in surrounding seawater. We suggest that these are symbiotic bacteria essential for healthy sponge holobionts.

**Acknowledgments** Sequencing was supported by a grant from the Ministry of Science and Higher Education of Russian Federation allocated to the Kurchatov Center for Genome Research (grant No. 075-15-2019-1659). Bioinformatic analysis was supported by the Agreement with the Ministry of Science and Higher Education of Russian Federation No. 075-15-2019-1661 and the Russian Foundation for Basic Research (project No. 20-34-90069).

## P3

### Identical Protein Group content and resequencing statistics as a naive metric of biological

### assembly quality: An evaluation study and draft tool implementation

#### Yury V. Malovichko^1,2*^, Ruslan O. Alagov^1,3^, Anton E. Shikov^1,2^, Alexander K. Predeus^4^, Anton A. Nizhnikov^1,2^, Kirill S. Antonets^1,2^

##### ^1^Laboratory for Proteomics of Supra-Organismal Systems, All-Russia Research Institute of Agricultural Microbiology, Pushkin, Saint Petersburg, Russia; ^2^Faculty of Biology, Saint Petersburg State University, Saint Petersburg, Russia; ^3^Institute of Technology, Mechanics and Optics, Saint Petersburg, Russia

## Bioinformatics Institute, Saint Petersburg, Russia

*Correspondence: Yury V. Malovichko yu.malovichko@arriam.ru

**Background** With the advance of next-generation sequencing techniques, repeated sequencing of already sequenced species has become a common procedure. Prokaryotes are more subjected to genome resequencing due to their modest genome size, the number of accessions for eukaryotic genome and transcriptome assemblies is also steadily growing. Despite obvious biological variations between different accessions, actual protein-encoding sequence content of any species is constrained by its evolutionary history and ecological niche, with the accuracy of these constraints’ estimation growing with each new assembly added. In this work we pried whether the species-specific content of entities from the NCBI Identical Protein Groups (IPG) database may be used as a naive metric for both assembly quality assessment and a taxonomic sanity check. We also present a piece of software, IPGQC, which implements the proposed pipeline.

**Methods** 227 bacterial species having more than ten genome assemblies of the highest completeness and adopted by RefSeq database were used for database construction. For the 12,624 assemblies selected, a total of 7,677,264 IPG representative sequences were pulled from NCBI IPG and used for database construction. For tool benchmarking, nine bacterial sequencing accessions having both Illumina and Oxford Nanopore Technologies reads available were selected for further reassembly with varying assembly (Raven, Flye, Unicycler, HybridSPAdes) and polishing (Medaka, Pilon) strategies. The assemblies were annotated using Prokka and underwent IPG content assessment against the database described for total IPG content and consistency with the scrutinized species.

**Results** IPGQC is currently implemented in pure Go language, while supporting scripts for custom database building written in Linux Shell. The suite is distributed alongside with the database used for tool benchmarking and can be found at https://github.com/ReverendCasy/IPGQC. The initial results demonstrated a steady growth of IPG content with the increase in assembly quality, but the exact numbers varied also according to the number of resequencing projects available for respective taxa.

**Conclusion** IPGQC is a fast and robust tool for preliminary quality assessment of genome assemblies which helps to choose the best genome assembly strategy and is also capable of draft taxonomic assignment.

**Acknowledgments** This work was supported by the Ministry of Science and Higher Education of the Russian Federation in accordance with agreement № 075-15-2020-920 date November 16, 2020, on providing a grant in the form of subsidies from the Federal budget of Russian Federation. The grant was provided for state support for the creation and development of a World-class Scientific Center “Agrotechnologies for the Future”.

## P4

### A pipeline for building mitochondrial genome supermatrices for partitioned phylogenetic analysis of fish

#### Sergei V. Turanov^1,2*^

##### ^1^A.V. Zhirmunsky National Scientific Center of Marine Biology, Far Eastern Branch, Russian Academy of Sciences, Vladivostok, Russia, 690041; ^2^Chair of Water Biological Resources and Aquaculture, Far Eastern State Technical Fisheries University, Vladivostok, Russia, 690087

*Correspondence: Sergei V. Turanov sturcoal@mail.ru

**Background** Complete mitochondrial genome sequences have proven to be particularly useful for phylogenetic reconstructions. They are highly informative, making it possible to infer phylogenies and date divergence, even among relatively deep and distant nodes. The use of mitochondrial genomes in partition-based phylogenetic reconstructions is associated with a number of challenges that require the need for correct annotation, parsing, and alignment of individual genome fragments, as well as concatenation into an annotated supermatrix. This paper presents the FishMitoPipe, a pipeline for parsing an annotated fish genome in order to perform an accurate phylogenetic analysis based on all (including the control region and ribosomal subunits) or a selection of annotated genome fragments.

**Methods** The pipeline is written in Shell and uses the pyfaidx package (Python), as well as the programs seqtk, MAFFT and phyutility. It uses the output from the fish mitochondrial genome annotation produced by MitoAnnotator, and implements several scripts that consecutively i) converts the mitochondrial sequences to a non-interleaved format, ii) removes intervals and spaces in the names, iii) renames the duplicated fragments, iv) splits the files of each sample into individual fragments, v) creates matrices for each fragment, vi) aligns them if needed, and vii) compiles the supermatrix for partitioned phylogenetic analysis.

**Results** The pipeline was tested on the Ubuntu 20.04.2 under the Linux Subsystem for Windows on different fish groups. No artefacts were produced during testing, however the pipeline is sensitive to the input file names and hence the respective recommendations in README file should be considered.

**Conclusions** This pipeline is comparable to similar tools (Phylomito, AnnotationBustR, PhyloSuite), but was developed and applied to extract and study the control region of fish mitochondrial DNA. The pipeline is available through https://github.com/Sturcoal/FishMitoPipe

**Acknowledgements** This research was partially supported by Ministry of Science and Higher Education of the Russian Federation (agreement number 075-15-2020-796, grant number 13.1902.21.0012).

## P5

### Computational inference of metabolic pathways and phenotypes in metagenome assembled genomes

#### Marat D. Kazanov^1,2^, Semen A. Leyn^3^, Dmitry A. Rodionov^1,3*^

##### ^1^A.A. Kharkevich Institute for Information Transmission Problems, Moscow, Russia; ^2^Skolkovo Institute of Science and Technology, Moscow, Russia; ^3^Sanford Burnham Prebys Medical Discovery Institute, La Jolla, CA, USA

*Correspondence: Dmitry A. Rodionov rodionov@sbpdiscovery.org

**Background** Metagenomic sequencing allows to study microbial communities, human microbiota, based on 16S rRNA gene amplicon data. We have developed a new approach to calculate metabolic capabilities of community, e.g. nutrient requirements, utilization, and production capabilities [1]. The approach is based on integration of binary encoded metabolic phenotypes inferred from expert biocuration and in silico genomic reconstruction across a large collection of microbial genomes. Binary phenotypes, where ‘1’ stands for the presence of a particular metabolic capability and ‘0’ stands for its absence, were obtained by mcSEED-based metabolic reconstruction of pathways using their complete genomes. A binary phenotype matrix developed in our previous studies, captures utilization and/or production of > 100 metabolites including carbon sources, essential nutrients, and fermentation end-products spanning > 2600 reference genomes of human gut microbiome. In other studies, we showed examples of usability of binary metaboli phenotypes for predictive functional characterization of metagenomic datasets [2, 3].

**Methods** For binary metabolic phenotype inference, we used three complementary approaches and consensus phenotype rules. The key input data for all three approaches included: (i) a set of > 70 metabolic pathways curated for a collection of 2856 HGM reference genomes in SEED genomic platform providing patterns of presence/absence of protein-encoding genes assigned functional roles that contribute to in silico reconstructed metabolic pathway variants and respective phenotypes; and (ii) a Reference BPM (Binary Phenotype Matrix) capturing inferred 1/0-values for > 100 metabolic phenotypes derived from these subsystems. First approach uses the Decision Tree method and the R’s Rpart library to elucidate gene patterns in reference genomes corresponding to a functional pathway variant and develop explicit logical Pathway Rules (PR) to assign binary phenotypes. Second approach uses the same collection of gene patterns to train > 30 Machine Learning (ML) methods available from the Caret package using one-leave-out approach. We selected Random Forest as a method with the best performance on our dataset. The prediction accuracy, i.e. a number of correctly predicted phenotypes for a particular subsystem, was used as a prediction quality metric. Ultimate ML models were constructed with the adjustment of the method’s parameters using the grid search and used to predict binary (1/0) values for the same set of > 100 phenotypes. Third approach allowed binary phenotype inference based on comparison of gene patterns within narrowly defined phylogenetic Neighbor Groups (NGs). This approach is based on the observed (and well-anticipated) limited variations of such patterns between closely related species and strains. We compiled a set of NGs comprised of target genomes and closely related reference genomes with Mash/MinHash distance ≤ 0.1. Within each NG and for each metabolic pathway, we tentatively assigned a binary phenotype value for a given genome based on the NG genome with the closest matching gene pattern using Hamming distance as a metric for binary comparison of gene patterns. At the final stage, we developed rules to assign consensus phenotypes and confidence categories based on the combination of the results of PR-, ML, and NG-based methods.

**Results** The number of isolated and sequenced human gut microorganisms with available genomes is rapidly growing. Uncultured bacteria with Metagenome Assembled Genomes (MAGs) expand substantially the phylogenetic diversity of human gut microbiome species. We developed a computational pipeline for automated propagation of curated metabolic phenotypes from the reference binary phenotype matrix over new microbial genomes from represented phylogenetic groups. First, we built a rule-based phenotype assignment algorithm using the genomic distribution of orthologs and a set of phenotype rules obtained by the formal decision tree approach. Second, we trained several machine learning models on reference sets of genes and phenotypes comprising > 70 metabolic pathways in > 2600 reference human gut bacterial genomes. Third, we implemented the neighbor-based approach that assign a phenotype based on variability of metabolic phenotypes and genes within a group of phylogenetically close neighbors (> 90% similarity) from the reference genomic collection. Combination of all three approaches allowed us to assign the consensus binary phenotypes with > 99% accuracy.

**Conclusions** We applied this combined genome annotation pipeline to three public collections of > 5500 human gut bacterial isolate genomes [4–6], as well as to > 11,000 high-quality MAGs from a recent study [7] to obtain expanded binary phenotypes covering a larger phylogenetic space of human gut microbial strains. We analyzed the phenotype variability in the obtained expanded genomic collection at the species level and report individual phenotypes and species with the highest variability. Top variable phenotypes are involved in carbohydrate utilization and biosynthesis of some vitamins.

**Acknowledgements** This research was supported by NIH (DK030292-35) and Russian Science Foundation (19-14-00305). We are grateful to Andrei Osterman for useful discussions and encouragements.

**References**

1. Rodionov DA, Arzamasov AA, Khoroshkin MS, Iablokov SN, Leyn SA, Peterson SN, Novichkov PS, Osterman AL. Micronutrient requirements and sharing capabilities of the human gut microbiome. Front Microbiol. 2019; 10:1316.
2. Iablokov SN, Novichkov PS, Osterman AL, Rodionov DA. Binary metabolic phenotypes and phenotype diversity metrics for the functional characterization of microbial communities. Front Microbiol. 2021; 12:653314.
3. Iablokov SN, Klimenko NS, Efimova DA, Shashkova T, Novichkov PS, Rodionov DA, Tyakht AV. Metabolic phenotypes as potential biomarkers for linking gut microbiome with inflammatory bowel diseases. Front Mol Biosci. 2021; 7:603740.
4. Forster SC, Kumar N, Anonye BO, Almeida A, Viciani E, Stares MD, Dunn M, Mkandawire TT, Zhu A, Shao Y, Pike LJ, Louie T, Browne HP, Mitchell AL, Neville BA, Finn RD, Lawley TD. A human gut bacterial genome and culture collection for improved metagenomic analyses. Nat Biotechnol. 2019; 37:186–192.
5. Poyet M, Groussin M, Gibbons SM, Avila-Pacheco J, Jiang X, Kearney SM, Perrotta AR, Berdy B, Zhao S, Lieberman TD, Swanson PK, Smith M, Roesemann S, Alexander JE, Rich SA, Livny J, Vlamakis H, Clish C, Bullock K, Deik A, Scott J, Pierce KA, Xavier RJ, Alm EJ. A library of human gut bacterial isolates paired with longitudinal multiomics data enables mechanistic microbiome research. Nat Med. 2019; 25:1442–1452.
6. Zou Y, Xue W, Luo G, Deng Z, Qin P, Guo R, Sun H, Xia Y, Liang S, Dai Y, Wan D, Jiang R, Su L, Feng Q, Jie Z, Guo T, Xia Z, Liu C, Yu J, Lin Y, Tang S, Huo G, Xu X, Hou Y, Liu X, Wang J, Yang H, Kristiansen K, Li J, Jia H, Xiao L. 1,520 reference genomes from cultivated human gut bacteria enable functional microbiome analyses. Nat Biotechnol. 2019; 37:179–185.
7. Almeida A, Mitchell AL, Boland M, Forster SC, Gloor GB, Tarkowska A, Lawley TD, Finn RD. A new genomic blueprint of the human gut microbiota. Nature. 2019; 568:499–504.

## P6

### Genome-wide detection of positive selection in new African cheetah assembly

#### Alina Fyodorova^1,*^, Anna Zhuk^1^, Pavel Dobrynin^1^

##### ^1^ITMO University, 49 Kronverkskiy Prospekt, St. Petersburg, 197101, Russia

*Correspondence: Alina Fyodorova lnfyodorova@gmail.com

**Background** Genome-wide scans for genes that have been targeted by natural selection can provide insight into the dynamics of genome evolution, the genetic basis of differences between species, explain adaptations to local environmental conditions and the functions of individual genes. There are two types of natural selection in biological evolution: positive (Darwinian) selection promotes the spread of beneficial alleles, and negative (or purifying) selection hinders the spread of deleterious alleles. PAML is a package of programs for phylogenetic analyses of DNA and protein sequences using maximum likelihood. The package has a rich repertoire of evolutionary models implemented, which include a model that classifies the different codon sites into different categories: evolving under purifying selection, neutral evolution and positive selection. However, this model is highly sensitive to errors in sequence alignment and therefore alignments have the potential to be highly enriched for false positives.

**Methods** Efficient algorithms are needed to mitigate false positives. One of such algorithms is extensive filtering of alignments using multiple filtering techniques: aligning orthologous genes with the PRANK algorithm, masking of inconsistent residues using GUIDANCE2, further masking for inconsistent regions using two different schemes, like select conserved blocks of sequence with Gblocks or a sliding window approach SWAMP to mask regions of the alignment with excessive amino acid changes.

**Results** We have implemented this algorithm in the GScour pipeline, making it possible to find genes under positive selection from genome data. All pipeline steps are accompanied by logging, automatic format conversion, sorting into different species groups of interest, construction of auxiliary files, calculation of significance using the likelihood ratio test, Bonferroni's and false discovery rate corrections, and summarizing results in tables. The GScour pipeline was able to replicate and clarify results from the previous study [1] of the African cheetah genome. This work was supported by RFBR project number 20-34-70055.

**Conclusions** The new pipeline is effective in detecting false positives and reproducing results from the previous study, although it is very sensitive to the method used for identification of orthologous genes.

**Reference**

1. Dobrynin, P., Liu, S., Tamazian, G. et al. Genomic legacy of the African cheetah, Acinonyx jubatus. Genome Biol 16, 277 (2015).

## P7

### Design and implementation of Telegram bots for biomedical research support

#### Ekaterina I. Smirnova^1,2*^, Kirill S. Antonets^1,3*^

##### ^1^St. Petersburg State University, St. Petersburg, Russia; ^2^Pavlov First Saint Petersburg State Medical University, St. Petersburg, Russia; ^3^All-Russia Research Institute for Agricultural Microbiology (ARRIAM), Pushkin, St. Petersburg, Russia

*Correspondence: Ekaterina I. Smirnova kate.smirnova.2016@gmail.com; Kirill S. Antonets k.antonets@arriam.ru

**Background** In the world of science, the efficiency of work largely depends on the data transmission speed and receipt of information. With mobile devices in hand, anyone can find answers to almost any question from everyday life within a few seconds. And one of the most useful and easy to use mobile applications are messengers. Telegram messenger is a technologically advanced messenger, useful and popular, with rich and extendable ecosystems consisting of bots for small and convenient tasks. Telegram bots are widely used for various purposes and can serve as a good complement to biomedical researchers. The NCBI houses a series of databases related to biotechnology and biomedicine and is an important resource for bioinformatics and biomedicine. The goal of the project was to build a Telegram bot for simple and intuitive work with the NCBI database, especially with the PubMed database. The developed Bot is aimed to assist retrieving information from NCBI databases and executing simple bioinformatic tasks with mobile devices on feet.

**Methods** The Telegram bot was implemented in Python programming language, using the official library for Telegram Bot API and The Entrez Global Query Cross-Database Search System. The sequence alignment and phylogenetic tree construction features were added using MAFFT [1] and raxml-ng [2] programs.

**Results** The developed Bot provides users the possibility to retrieve abstracts for 20 articles from the PubMed based on the keyword or author name search. Also by entering the accession number of the nucleotide or protein sequence, the user can download a document with a record of this sequence in fasta format. And finally, by sending a document with several sequences in FASTA format, the user will receive a document with a built phylogenetic tree based on the sequence's alignment.

**Conclusions** The developed product will reduce the time spent on finding the necessary information for doctors, biomedical researchers, medical students and general public and provide the possibility to work with biological data directly in messenger interface using mobile devices.

**References**

1. Katoh, Kazutaka, et al. "MAFFT: a novel method for rapid multiple sequence alignment based on fast Fourier transform." *Nucleic acids research* 30.14 (2002): 3059–3066.
2. Kozlov, Alexey M., et al. "RAxML-NG: a fast, scalable and user-friendly tool for maximum likelihood phylogenetic inference." *Bioinformatics* 35.21 (2019): 4453–4455.

## P8

### Applications of coarse grained enhanced molecular dynamics for antibody structure modelling

#### Valentina Maslova^1,2*^, Andrey Golovin^1,2,3^

##### ^1^Faculty of Bioengineering and Bioinformatics, Lomonosov Moscow State University, Moscow, Russia; ^2^Sirius University of Science and Technology, Sochi, Russia; ^3^Shemyakin-Ovchinnikov Institute of Bioorganic Chemistry of the Russian Academy of Sciences, Moscow, Russia

*Correspondence: Valentina Maslova val_ma@fbb.msu.ru

**Background** Antibodies are proteins that recognize the antigen surfaces during the adaptive immune response. Continued exploitation of antibodies for therapeutic purposes relies on efficient ways to develop them. Antibody structures may be useful during drug development, allowing the implementation of rational design. The most challenging part of the antibody structure prediction is to model the H3 loop, which is also often important for antigen binding. The majority of H3 loop prediction approaches are based on computationally expensive decoy generation. The challenge in such ab initio modelling remains in selection of best generated loop models [1]. Coarse grained (CG) models in molecular modelling are valuable for probing the time scales of systems beyond what is achievable with full-atom models. The recently released CG forcefield Martini3 [2] with an expanded ability to include interactions such as hydrogen bonding and electronic polarizability may be useful for antibody structure modelling.

**Methods** We aimed to demonstrate the potential application of CG molecular dynamics (MD) for the task of H3 loop conformation sampling. We used a pair of apo- and antigen-bound antibody Fab-fragment structures of ferrochelatase antibody 7G12 with differing H3 loop conformations (PDB IDs 1NGZ, 1N7M). The molecular dynamics simulation based on CG forcefield Martini3 was set up starting from an antigen-bound structure with antigen removed. We used Hamiltonian replica-exchange (HREX) MD with the H3 loop assigned to be the hot region to enhance conformational sampling.

**Results** The overall Fab structure remained stable in all HREX replicas. The most frequent H3 loop conformations present in the trajectory resembled the antigen-bound structure. The conformation of the H3 loop present in the unbound antibody crystal structure was observed during the HREX MD in cold replicas, yet it is less stable than the one of the starting bound-like structure. However the 7G12 apo-structure may be a poor reference for the unbound H3 loop conformation, since there is a contact between the tail region of the crystal symmetry mate Fab and the H3 loop in it [3].

**Conclusion** CG MD with Martini3 allows to sample H3 loop conformations of 7G12, tending to favor the one adapted for antigen binding, thus proving the CG MD suitable for antibody molecular modelling.

**Acknowledgements** The reported study was funded by RFBR, project number 19-34-51043, and supported by Russian Science Foundation Grant 21-74-20113. The research is carried out using the shared research facilities of HPC computing resources at Lomonosov Moscow State University [4].

**References**

1. Norman RA, Ambrosetti F, Bonvin AMJJ, Colwell LJ, Kelm S, Kumar S, Krawczyk K. Computational approaches to therapeutic antibody design: established methods and emerging trends. Brief Bioinform. 2020; 21(5):1549–1567.
2. Souza PCT, Alessandri R, Barnoud J, Thallmair S, Faustino I, Grünewald F, Patmanidis I, Abdizadeh H, Bruininks BMH, Wassenaar TA, Kroon PC, Melcr J, Nieto V, Corradi V, Khan HM, Domański J, Javanainen M, Martinez-Seara H, Reuter N, Best RB, Vattulainen I, Monticelli L, Periole X, Tieleman DP, de Vries AH, Marrink SJ. Martini 3: a general purpose force field for coarse-grained molecular dynamics. Nat Methods. 2021; 18(4):382–388.
3. Fernández-Quintero ML, Kraml J, Georges G, Liedl KR. CDR-H3 loop ensemble in solution—conformational selection upon antibody binding. MAbs. 2019; 11(6):1077–1088.
4. Voevodin V, Antonov A, Nikitenko D, Shvets P, Sobolev S, Sidorov I, Stefanov K, Voevodin V, Zhumatiy S. Supercomputer Lomonosov-2: Large Scale, Deep Monitoring and Fine Analytics for the User Community. Supercomput Front Innov. 2019; 6(2):4–11.

## P9

### Conservative blocks in C-terminal regions of 3-D Cry toxins exhibit amyloidogenic properties

#### Anton E. Shikov^1,2*^, Yury V. Malovichko^1,2^, Ruslan O. Alagov^1,3^, Anton A. Nizhnikov^1,2^, Kirill S. Antonets^1,2^

##### ^1^Laboratory for Proteomics of Supra-Organismal Systems, All-Russia Research Institute of Agricultural Microbiology, Pushkin, Saint Petersburg, Russia; ^2^Faculty of Biology, Saint Petersburg State University, Saint Petersburg, Russia; ^3^Institute of Technology, Mechanics and Optics, Saint Petersburg, Russia

*Correspondence: Anton E. Shikov a.shikov@arriam.ru

**Background** A spore-forming bacterium, *Bacillus thuringiensis*, is a source of efficient and specific insecticidal moieties. Of these virulence factors, the most abundant and frequently used class is represented by the so-called 3-D Cry toxins. While these toxins share a rather conservative structure, bearing three domains exerting pesticidal activity, they also possess flanking sequences. Both N- and C-terminal regions are processed when the toxin enters the host gut; however, the latter has been demonstrated to determine the formation of crystals. Nevertheless, the mechanism governing crystallization as well as the diversity of flanking regions are yet to be elucidated.

**Methods** 3-D Cry toxin sequences have been retrieved from all publicly available data sources, including the Genbank database, *Bt*- genome assemblies. Flanking regions were obtained using domain coordinates provided by CryProcessor [1], a tool specifically designed for retrieving 3-D Cry toxins from genetic data. For revealing conservative blocks reflecting putative subdomains we developed a novel method based on building k-mer profile with hierarchical clustering followed by the reconstruction of cluster chains and deduplication using CD-HIT utility [2]. To predict amyloidogenic sites we have utilized two algorithms, Waltz [3] and SARP [4].

**Results** We summarized conservative blocks within these terminal parts of Cry toxin sequences and examined them for amyloidogenic characteristics possibly modulating the crystallization process. The number of reconstructed blocks reached 204 and 100 for C- and N-terminal sequences, respectively. Noteworthy, C-terminal regions fell into two clusters based on their lengths. The frequency analysis revealed a significant correlation between the number of blocks and the total sequence length for C-terminal regions, which corroborated length-based clustering: longer sequences possessed from 3 to 5 blocks on average, while the short ones carried 1–2 blocks. The N-terminal region did not display such a pattern. For each unique block, the overall abundance among analyzed toxins and the number of amyloidogenic sites were summarized. While in the N-terminal region, amyloidogenic sites were only sporadically detected by Walts, C-terminal sequences possessed two conservative blocks carrying amyloidogenic signals found in more than half of sequences.

**Conclusion** The results obtained demonstrate an immense structural diversity of the C-terminal region of 3-D Cry toxins with regard to the overall region length, number, and variety of subdomains. Moreover, amyloidogenic sites found in conservative blocks may indicate that the C-terminal region can modulate crystallization through local protein aggregation in these sites.

**Acknowledgments** This study was supported by the Russian Science Foundation (20-76-10044).

**References**

1. Shikov, A.E.; Malovichko, Y.V.; Skitchenko, R.K.; Nizhnikov, A.A.; Antonets, K.S. No More Tears: Mining Sequencing Data for Novel Bt Cry Toxins with CryProcessor. Toxins, 2020, V. 12, 204. https://doi.org/10.3390/toxins12030204.
2. Limin Fu, Beifang Niu, Zhengwei Zhu, Sitao Wu and Weizhong Li, CD-HIT: accelerated for clustering the next generation sequencing data. Bioinformatics, 2012, V. 28 (23), 3150–3152. https://doi.org/10.1093/bioinformatics/bts565.
3. Maurer-Stroh, S., Debulpaep, M., Kuemmerer, N. et al. Exploring the sequence determinants of amyloid structure using position-specific scoring matrices. Nat Methods, 2010, V. 7, 237–242. https://doi.org/10.1038/nmeth.1432.
4. Antonets KS, Nizhnikov AA. SARP: A Novel Algorithm to Assess Compositional Biases in Protein Sequences. Evol Bioinform Online, 2013, V. 9, 263–273. https://doi.org/10.4137/EBO.S12299.

## P10

### Metagenomes of the microbial cellulolytic communities on various substrates

#### Grigory G. Gladkov^1,2*^, Tatyana O. Lisina^1^, Anastasiia K. Kimeklis^1, 2^, Arina A. Kichko^1, 2^, Alexey M. Afonin^1^, Evgeny E. Andronov^1,2,3^

##### ^1^All-Russian Research Institute for Agricultural Microbiology, Pushkin, Saint-Petersburg, 196608, Russia; ^2^St. Petersburg State University, Saint-Petersburg, 199034, Russia; ^3^V.V. Dokuchaev Soil Science Institute, Moscow, 119017, Russia

*Correspondence: Grigory G. Gladkov ruginodis@gmail.com

**Background** Studies of microorganisms with the ability to degrade cellulose are of both theoretical and applied interest. Two approaches dominate in this field—the search for genes associated with efficient cellulose degradation and ecological studies of natural communities associated with cellulose degradation. Ecological studies of this kind usually use metabarcoding methods for taxonomy analysis, but here we have combined them with analysis of the complete metagenome using long-read sequencing methods.

**Methods** Microbial communities decomposing cellulose on various substrates (straw, litter, sawdust) with the addition of a biopreparation based on active peat soil were isolated on selective medium. We acquired four sustainable communities and investigated their metagenomes. The 16S rRNA libraries for bacteria and ITS for fungi were sequenced using Illumina technology. We used compositional data analysis approaches (phylogenetic isometric log-ratio transformation) to describe the differences between communities. In addition, metagenomic sequences were assembled for each community using Oxford Nanopore technology. After polishing and assembly, the carbohydrate-active (CA) enzyme families were identified from the CAZypedia database (HMMER, Hotpep, and DIAMOND algorithms were used).

**Results** According to the amplicon sequencing, each community is represented by 80 to 150 different bacteria. Taxonomic attribution by metagenomic data and amplicon sequencing was consistent. In this case, we can speak of a qualitative rather than quantitative match. Communities from the same substrate have similar taxonomic profiles, from different substrates they differ greatly. 10 complete genomes were isolated from the assembly. 2582 matches of CA enzymes were found in the metagenomes. The taxonomic profile of the contigs in which CA enzymes were annotated is consistent with the 16S taxonomy results for the communities. Despite the differences in the taxonomy, there were no strong differences in the representation of CA enzymes families in the metagenomes of communities.

**Conclusions** The studied cellulolytic communities are a conglomerate of microorganisms, often individually lacking the functional genes necessary for full decomposition of cellulose-based complex substrates. At the same time, the communities comprise a whole complex of separate clusters of often closely related organisms, whose individual functional role remains unclear to us. It has been shown that the substrate specificity of communities appears mainly at the taxonomic level. When we attempted to compare metagenomes at the level of functional gene families, no differences were found.

**Acknowledgments** The work is supported by Russian Science Foundation grant named RSF 18-16-00073.

## P11

### Methylation patterns of *Rhizobium leguminosarum* genome change in terminally differentiated cells

#### Alexey Afonin^1,2*^, Gribchenko Emma^1^, Evgeny Zorin ^1,2^, Aksenova Tatyana^1^, Anton Sulima^1^, Daria Romanyuk^1^, Vladimir Zhukov^1,2^

##### ^1^All-Russia Research Institute for Agricultural Microbiology, Saint-Petersburg, Russia; ^2^Sirius University of Science and Technology, Sochi, Russia

*Correspondence: Alexey Afonin aafonin@arriam.ru

**Background** Bacteria of the Rhizobia group possess a unique ability to form symbiotic relationships with the plants of the Fabaceae family. During the formation of symbiosis both the organisms synchronise, which results in the formation of nitrogen-fixing nodules. Bacteria terminally differentiate into bacteroids—a nitrogen fixing form, uncapable of leaving the nodule. Although the polyploidy of bacteroid forms is a well-established fact [1], not much is known about other forms of DNA modifications in pea-rhizobia symbiosis. Objective of this study was to investigate the changes in the DNA of *Rhizobium leguminosarum* RCAM1026 strain occurring during terminal differentiation in nodules of garden pea (*Pisum sativum* L.).

**Methods** The free-living cell culture of the strain RCAM1026 [2] was harvested after three days of growth at 28 °C in TY media. The bacteroid DNA was isolated from bacteroids, which were isolated from nodules formed on the roots of *Frisson* cultivar plants four weeks after inoculation. MinION sequencer (Oxford Nanopore) was used for genome sequencing. Reads were basecalled using guppy caller (v. 4.4.2), the genome was assembled with flye [3] (3.8). Structural variations were analysed using MUMmer (v 4.0.0) [4]. Nanodisco (v. 1.0.2) [5] was used to investigate the differences in methylation between the conditions.

**Results** The genome consisted of 5 circular replicons, with no structural variations between bacterial forms detected. The symbiotic plasmid showed two times lower coverage in bacteria; in the bacteroids the coverage of the chromosome was two times that of the rest replicons, with all plasmids having very similar coverage. Two distinct methylation patterns were found in free-living culture: GANTC and GGCGCC, only one distinct methylation pattern was found in the bacteroid genome: GGCGCC. The GGCGCC site seem to represent a restriction enzyme site, previously not described for *R. leguminosarum*. The GANTC motif was much less prevalent in bacteroids, than in free-living culture.

**Conclusions** No genome structural differences were observed in bacteroids. The changes in methylation of GANTC site may be one of the mechanisms switching the bacteria to the terminally differentiated state, and may result in the observed replicon abundance variations.

**Acknowledgements** This work was supported by the RFBR [research project № 19-316-51014].

**References**

1. Maróti G, Kondorosi É. Nitrogen-fixing Rhizobium-legume symbiosis: are polyploidy and host peptide-governed symbiont differentiation general principles of endosymbiosis? Front Microbiol. 2014;5. https://doi.org/10.3389/FMICB.2014.00326.
2. Afonin A, Sulima A, Zhernakov A, Zhukov V. Draft genome of the strain RCAM1026 Rhizobium leguminosarum bv. viciae. Genomics Data. 2017;11:85–6. https://doi.org/1016/j.gdata.2016.12.003.
3. Kolmogorov M, Yuan J, Lin Y, Pevzner PA. Assembly of long, error-prone reads using repeat graphs. Nat Biotechnol. 2019;37(5):540–6. https://doi.org/10.1038/s41587-019-0072-8.
4. Marçais G, Delcher AL, Phillippy AM, Coston R, Salzberg SL, Zimin A. MUMmer4: A fast and versatile genome alignment system. Darling AE, editor. PLOS Comput Biol. 2018;14(1):e1005944. https://doi.org/10.1371/journal.pcbi.1005944.
5. Tourancheau A, Mead EA, Zhang X-S, Fang G. Discovering multiple types of DNA methylation from bacteria and microbiome using nanopore sequencing. Nat Methods. 2021;18(5):491–8. https://doi.org/10.1038/s41592-021-01109-3.

## P12

### Phylogenetic Relationships of Ascomycetes Opine Synthases

#### Sofia Sokornova^1*^, Tatiana Matveeva^1^

##### ^1^Department of Genetics and Biotechnology, St. Petersburg State University, Saint Petersburg, 199034, Russia

*Correspondence: Sofia Sokornova svsokornova@vizr.spb.ru

**Background** Opines are low molecular weight compounds that are synthesized in organisms of different taxa. They owe their name to octopine, first discovered in cephalopods, where it is formed during the anaerobic oxidation of glucose by octopine dehydrogenase. At the moment, the most famous are opines of agrobacterial origin. These are strain-specific metabolites that are synthesized in the crown galls and hairy roots induced by these bacteria and metabolized by the same bacterial strains. There are agrobacterial opine genes transferred into plant genomes million years ago. It is believed that their products play a role in the regulation of plant–microbe interactions [1]. Genes annotated as opine synthases were found also in fungi. The aim of our study is to analyze the phylogenetic relationships of the ascomycete opine synthases.

**Methods** At the first step by Maximum Likelihood method [2] we analyzed the relationship of opine-synthases (octopine/nopaline dehydrogenase family proteins) from bacterial and eukaryotic organisms. It was shown that sequences of bacteria, naturally transgenic plants, and ascomycetes form common cluster independent from marine invertebrates. At the second stage of our study, we carried out tblastn search for opine synthases genes as described earlier [3] against all known sequenced genomes of ascomycetes and performed their phylogenetic studies by Maximum Likelihood method based on the GTR model [2]. Initial tree(s) were obtained automatically by applying Neighbor-Join and BioNJ algorithms to a matrix of pairwise distances estimated using the Maximum Composite Likelihood, then selecting the topology with superior log likelihood value in MEGA7 [4].

**Results** In the genomes of some Pezizomycetes, Eurotiomycetes; Leotiomycetes; Saccharomycetes; Sordariomycetes; Blastocladiomycetes; Mucoromycetes homologues of ags/chs, ocs/vis, mis were found. Phylogenetic analyses show mosaic arrangement of species containing different opine synthases on the phylogenetic tree.

**Conclusions** It suggests the role of horizontal transfer in the spread of these sequences in the fungi or evolution from a common ancestral sequence by losing part of it. Since among fungi there are many species that enter symbiosis with plants and other microbes, then the idea of the role of opines in the regulation of plant–microbe interactions looks even more intriguing.

**Acknowledgements** This work was supported by grant from the RSF 21-14-00050.

**References**

1. Matveeva TV, Otten L. Opine biosynthesis in naturally transgenic plants: Genes and products. Phytochemistry. 2021; 189: 112813.
2. Nei M, Kumar S. Molecular Evolution and Phylogenetics. 2000. Oxford University Press, New York.
3. Matveeva TV, Otten L. Widespread occurrence of natural genetic transformation of plants by Agrobacterium. PMB 2019; 101(4–5):415–437.
4. Kumar S, Stecher G, Tamura K. MEGA7: Molecular Evolutionary Genetics Analysis Version 7.0 for Bigger Datasets, Molecular Biology and Evolution. 2016; 33(7):1870–1874.

